# Successful Control of *Mycobacterium avium* Subspecies *paratuberculosis* Infection in a Dairy Herd within a Decade—A Case Study

**DOI:** 10.3390/ani14060984

**Published:** 2024-03-21

**Authors:** Karsten Donat, Esra Einax, Doreen Rath, Anne Klassen

**Affiliations:** 1 Animal Health Service, Thuringian Animal Diseases Fund, Victor-Goerttler-Straße 4, 07745 Jena, Germany; eeinax@thtsk.de (E.E.); aklassen@thtsk.de (A.K.); 2 Veterinary Clinic for Reproductive Medicine and Neonatology, Justus-Liebig-University Giessen, Frankfurter Straße 106, 35392 Giessen, Germany; 3 Argar T&P GmbH Mockzig, 04470 Altenburg, Germany; doreen.rath@agrargmbhmockzig.de

**Keywords:** Johne’s disease, voluntary control, faecal culture, ELISA, true prevalence, survival rate, milk yield

## Abstract

**Simple Summary:**

Paratuberculosis impacts animal welfare and the economic performance of dairy herds by causing reduced milk yield and carcass weight and premature culling. Due to the ability of the infectious agent *Mycobacterium avium* subsp. *paratuberculosis* (MAP) to survive in the environment for a long time, the limited diagnostic accuracy of tests, and the long incubation period, successful elimination of MAP from a cattle herd is doubted. This study describes measures applied to control the disease in a 450-head dairy herd and, as the result of these measures, the progress of prevalence reduction to a level where the infectious agent was not detectable anymore in any individual sample and from the liquid manure of the herd. MAP shedders were detected by the bacteriological cultivation of individual faecal samples from each cow in annual intervals and MAP shedders were removed from the herd in a timely manner. Calves were kept outside the barn of adult cows and hygienic measures to minimize their MAP exposure were established. The results demonstrate that the elimination of MAP might be possible within 10 years. Voluntary control programs are effective in controlling paratuberculosis in closed herds by providing adequate diagnostic, logistic, and financial support for farmers.

**Abstract:**

This longitudinal case study provides an in-detail report of the process towards the elimination of *Mycobacterium avium* subsp. *paratuberculosis* (MAP) from a closed 450-head commercial dairy herd. In parallel, two diagnostic approaches were applied to all cows in annual intervals during 2012–2022: detection of MAP in individual faecal samples by bacteriological cultivation on solid medium and detection of MAP-specific antibodies by ELISA. For each annual sampling, the kappa coefficients for test agreement and the survival rates of MAP-positive and MAP-negative cows were calculated. Applying a multivariable linear regression model revealed a significantly lower fat-corrected 305-day milk yield for MAP-positive cows. The true prevalence of MAP shedders reduced from 24.2% in 2012 to 0.4% in 2019 and during 2020–2022, no MAP shedder was identified. Test agreement was generally low and bacteriological cultivation showed positive results earlier than the ELISA. In the first years of control, the survival of MAP shedders was longer than in the final stage. In conclusion, the elimination of MAP from a dairy herd might be feasible within a decade. Changes in the test agreement must be considered. Timely removal of MAP shedders, hygienic calf rearing, and colostrum supply are key for successful control.

## 1. Introduction

Paratuberculosis, or Johne’s disease, is a chronic granulomatous enteritis caused by an infection with *Mycobacterium avium* subspecies *paratuberculosis* (MAP). It affects mainly ruminants and impacts animal welfare, has direct and indirect economic costs, and arouses public health concerns. Animals with clinical signs suffer from diarrhoea, weight loss, and, finally, death [1]. In dairy cattle, economic losses are mainly caused by reduced milk yield and carcass weight and premature culling of the infected animals [2]. Depending on which costs were included, total annual economic losses were estimated to be between USD 21 and EUR 234 [3]. A link between MAP and several diseases in humans is widely discussed and numerous review articles have concluded that human exposure existed and the zoonotic potential of MAP cannot be ignored. Its impact on public health cannot yet be quantified due to relevant knowledge gaps in understanding its role in the development of human disease and, therefore, steps beyond the already existing programs for the reduction in economic losses and improvement of animal health could not be justified by public health authorities [4,5]. In other words, a reliance of public health authorities on animal health authorities to reduce the exposure of humans to MAP by controlling paratuberculosis in livestock was identified [3].

Paratuberculosis is prevalent globally; its prevalence and its impact will certainly increase if not dealt with by effective control measures [6]. The main important tools for control of paratuberculosis, as well as the management practices that should be implemented by the farmers and the limitations of those measures, have been known for a long time. The improvement of biosecurity within a herd to prevent new infections in youngstock, culling of clinical cases, identification and removal of subclinical cases (test-and-cull), and controlled buy-in of animals and environmental pasture management are the most important measures. Vaccination is mainly used in sheep and goats and is restricted by vaccine licensing in several countries [3]. Despite all this knowledge being available, an agreed international code for paratuberculosis, specifying the principles and methods of control ideally adopted by the O.I.E., is missed, to date, leading to the heterogeneity of existing control programs in different countries and, even worse, to the fragmentation of control activities within several countries [3]. Therefore, reports of the successful control of paratuberculosis are desired and needed, whether at the regional or herd level.

A valid report of the successful elimination of MAP from a herd with a high initial prevalence, as reported for goats [7], is still missing for cattle. The objective of this case study was to provide an in-detail report of the process toward the elimination of MAP from a closed 450-head commercial dairy herd in the framework of a voluntary regional control program and to provide an example for other dairy farmers and their veterinarians regarding how this goal can be achieved within a reasonable period.

## 2. Materials and Methods

### 2.1. Study Farm

The study herd was a commercial dairy herd keeping about 450 dairy cows during the last decade. Youngstock comprising about 420 female calves and heifers were raised on the farm. The barn was newly built in 2009 as a loose-house system with three rows of cubicles at each side of the feeding table, a separate calving pen, and a side-by-side milking system. Calves were kept in a separate barn outside the main building from the first day of their lives. Older youngstock were raised in another separate barn. 

The herd is owned by an agricultural company that employs 23 people and 2 apprentices. From the early sixties until the nineties of the former century, it was a cooperative farm that had been producing milk since the early seventies of the last century. The farm cultivates 850 hectares (ha) of arable land (loess clay and loess sand) and 150 ha of grassland in a low mountain foothills region with about 560 L/qm rain per year. In addition to the market crop production of winter barley, winter wheat, and winter rape (about 570 ha), the farm grows sugar beet (about 50 ha) and white cabbage (about 20 ha). The fodder basis for milk production is the cultivation of maize (170 ha) and agricultural grass (10 ha), as well as the mowing of the permanent grassland, to produce maize silage and grass silage. A biogas plant with a fermenter and a secondary fermenter of 1500 m^3^ each and a yearly production of 2150 MWh was put into operation in 2009. It utilizes mainly the liquid manure from the own cattle and neither solid manure from calves and youngstock nor manure from other farms. 

Other than milk production, the farm was active as a pedigree breeder, with all cows being pure-bred German Holstein. By purchasing genetically valuable cattle during the years 2002–2012, a total of 344 buy-in cattle originating from 36 other herds in four other German federal states were introduced into the herd. 

### 2.2. Initial Situation Regarding Paratuberculosis and Program Enrolment

The first cases with clinical signs suggestive of paratuberculosis were observed in 2011. The cases had been confirmed with the detection of MAP in individual samples. The farm enrolled in the voluntary Thuringian Paratuberculosis Control Program (TPCP) in 2012. The program was described in detail elsewhere [8,9]. In brief, the program relies on the identification and removal of MAP shedders by the annual testing of individual faecal samples from all cows for MAP, an on-farm veterinary risk assessment resulting in tailored recommendations for improving hygiene management, the consideration of the paratuberculosis status of buy-in cattle with respect to the status of the herd of origin, and the certification of herds as ‘non-suspect’ after three years without detection of MAP in the yearly testing of each cow. The costs for participation in the programme are to be borne by the farmer. The Thuringian Animal Diseases Fund grants financial aid for certain measures under the programme, i.e., half of the laboratory costs for testing the faecal samples, a subsidy of EUR 1 per sample for both drawing a blood sample and the costs of an ELISA test, and a subsidy of EUR 2 per sample for the veterinarian for taking the faecal samples. The granting of aid is conditional on compliance with the agreed measures. The measures are agreed upon in joint consultation with the farm, the herd’s veterinarian, the Animal Health Service (AHS) of the Thuringian Animal Diseases Fund, and the competent veterinary authority, and specified annually. Total costs of testing were calculated from the number of samples tested during each year in the laboratory of the AHS and the cost per test in the respective year. 

### 2.3. Sampling and Testing

As laid down in the TPCP, faecal samples were taken from each cow of the farm, for the first time in March 2012 until 2022 in annual intervals. Additionally, during the years 2012–2015, primiparous cows that calved after the sampling day were sampled in monthly intervals together with cows whose faecal samples had no valid results because of undesired growth. Results of the bacteriological cultivation of faecal samples (faecal culture, FC) were used to manage MAP shedders and their calves according to farm-specific recommendations (Level 3, herds with MAP shedder prevalence >3%) or to remove MAP shedders in a timely manner (Level 4, herds with MAP shedder prevalence ≤3%) [9]. Individual faecal samples were taken rectally using a new glove for each cow and stripping the faeces into a screwable plastic cup with a barcode. 

The testing of blood samples for MAP-specific antibodies in parallel to FC was the subject of a special agreement between the farm and the AHS and was performed from 2012 to 2020. For this purpose, blood samples taken to comply with the German regulation for Bovine Herpesvirus 1 (BHV1) surveillance were used. On the day of faecal sampling, whole-blood samples were taken from each cow older than 24 months by the farm’s veterinarian from the caudal tail vessels using an EDTA-Kabevette^®^ (Primavette V EDTA, Kabe Labortechnik GmbH, Nümbrecht-Elsenroth, Germany). 

In addition, on the same day as the individual samples were taken, samples from the biogas plant were taken. A sample of slurry from the sump before entering the first fermenter and one sample after the fermentation process from the final storage were collected. Samples were obtained using a small bucket attached to a rod. The material was collected in a clean bucket and, after mixing, approximately 100 mL was decanted into a sterile plastic cup with a screw cap. Furthermore, environmental samples were taken from the barn environment in November 2012 using the boot swap sampling approach, as described elsewhere [10]. Samples were taken from the walkways in the pens of the cows, the main alleyway to the waiting pen as well as the waiting pen in front of the milking parlour itself, the area in front of the calf igloos, and the sick cow pen. 

All faecal samples were transported straight to the laboratory of the AHS. Faecal samples were stored at 20 °C (±5 °C) until cultivation to avoid undesired bacterial and fungal growth. Blood samples were stored at 5 °C (±3 °C). 

On the day of arrival in the laboratory, whole-blood samples were centrifuged for 5 min 840× *g* (Rotanta TR 440, Hettich, Tuttlingen, Germany) and plasma was stored at 5 °C (±3 °C). A commercial ELISA system (ID Screen^®^ Paratuberculosis Indirect ELISA kit, ID Vet, Montpellier, France), which was licensed in Germany, was applied for the detection of MAP-specific antibodies according to the manufacturer’s instructions. Cut-offs recommended by the manufacturer were used. 

The FC of the individual faecal samples, the environmental samples, and the biomass samples was completed according to the official method, as laid down in the manual of diagnostic procedures of the Friedrich-Loeffler-Institut, the Federal Research Institute of Animal Health [11], and as previously described [12]. In short, 3 g of faeces or the eluate of a sampling sock was decontaminated with 30 mL of a 0.75% hexadecyl pyridinium chloride solution (HPC, Acros Organics, Fisher Scientific GmbH, Nidderau, Germany) and, after sedimentation, the supernatant (20 mL) was decanted and incubated for 48 h at room temperature and 0.2 mL of the sediment was inoculated on three slants of a commercial Herrold’s Egg Yolk Culture Medium (HEYM) with Mycobactin J (Becton Dickinson GmbH, Heidelberg, Germany). After incubation under aerobic conditions at 37 °C (±2 °C) for seven days (aerobic) and another eleven to fifteen weeks (anaerobic), slants were examined for mycobacterial growth every two weeks. Samples were characterized as MAP positive, if MAP was detected in the colony material by a conventional IS900 polymerase chain reaction (PCR) [13], or MAP negative. In case of the high amount of undesired bacterial and fungal growth on all three slants precluding a valid result of faecal culture, this outcome was communicated to the farmer together with the request to resubmit a sample. The level of shedding was categorized semi-quantitatively by the number of colony forming units (cfu) on the surface of the culture medium, with 1–10 cfu labelled as “low”, 11–50 cfu as “moderate”, 51–100 cfu as “high”, and >100 cfu as “very high”. Test results were reported as soon as a positive detection of MAP was made together with the information on which cows were high or very high shedders. 

In addition, from 2016 to 2019, a commercially available IS900-based real-time PCR protocol (Adiavet Paratb, Adiagene, Saint Brieuc, France) for direct detection of MAP DNA from faeces was applied to biomass samples after MAP DNA extraction from biomass samples using a commercial nucleic acid isolation kit (QIAamp DNA Mini Kit, Qiagen GmbH, Hilden, Germany) and pre-concentration of samples using Adiafilter (Adifil 100, Adiagene, Saint Brieuc, France). All kits were used according to the manufacturer’s instructions. A threshold of 37 Ct value was used to consider a sample positive for MAP DNA. The result was rated as inconclusive if the Ct value was in the range between higher than 37 and 40 or if the signal of the internal amplification control did not meet the reference range. 

### 2.4. On-Farm Management Measures for Mitigating the Risk of Spreading MAP 

A veterinary risk assessment was performed in June 2012. As a result, the following management measures were agreed on between the herd manager, the AHS, and the veterinary authority.

#### 2.4.1. Hygienic Measures Regarding Calving, Colostrum Supply, and Calf Rearing

- Separate calving pen for MAP shedders;
- Separation of calves from their dam immediately after calving;
- Keeping calves outside the premises for adult cows in individual igloos;
- Separate tools (broom, shovel, fork, bucket) for the calf area;
- Separate room for preparing milk for calves and cleaning the teat buckets, with entry only by calf carers and no entry with dirty boots;
- Careful milking of first colostrum using a clean separate milking machine, strictly avoiding faecal contamination of colostrum;
- In-line feeding of first colostrum (only from the own dam);
- Disposal of non-saleable milk from MAP-positive cows (1–5 days in milk) via liquid manure into the biogas plant;
- Mixed colostrum only from MAP-negative cows.

#### 2.4.2. Management Measures Regarding Animal Movement and the Handling of MAP Shedders

- Closing of the herd so that exclusively heifers raised on the farm were included in the dairy herd;
- Marking of MAP-positive cows and cows with MAP-specific antibodies using a separate neckband;
- Documentation of test results within the herd management software;
- Exclusion of MAP-positive cows and cows with MAP-specific antibodies from further breeding;
- Immediate culling of high or very high shedders.

Management measures were revised annually; however, since 2018, the female offspring of MAP-positive cows and cows with MAP-specific antibodies have been bred only to terminal sire to exclude this cow family from further pedigree breeding. 

Milk recording data (305-day fat-corrected milk, protein kg, fat kg) were gathered from a data backup of the internal management software (HerdeW, Version 5.12, dsp agrosoft GmbH, Ketzin, Germany), supplied by the farmer, and were analysed for the first five years of MAP control (2012–2016).

### 2.5. Statistical Analysis

Data recording and editing were performed using a Microsoft Excel spreadsheet (Version 2402, Microsoft Corporation, Washington, DC, USA). All further computations were made using R version 4.1.2 [14]. 

MAP shedder prevalence was calculated from the number of faecal-culture-positive cows in the respective sampling data by the number of cows with valid test results (positive or negative), without consideration of samples that were not analysable because of undesirable growth or other reasons. To calculate the seroprevalence of MAP-antibody-positive cows, samples with suspicious results were counted as negative. The Bayesian estimation of true prevalence was executed in R using the package prevalence [15].

The age on the day of the first positive test (FC and ELISA) was used to analyse the probability of getting a positive test result in relation to the age of the animals. This was estimated using the R package cmprsk [16].

On the individual cow level, kappa coefficients were calculated for the agreement between the test outcome of the FC (detection of the pathogen) and ELISA test (detection of MAP-specific antibodies) using the R package DescTools [17]. According to Grouven et al. [18], the following ranges were considered for the interpretation of the kappa coefficient: 0.81–1.00: excellent agreement, 0.61–0.80: moderate agreement, 0.41–0.60: substantial agreement, 0.21–0.40: low level of agreement, <0.20: slight level of agreement. Not valid results from FC were rated as missing. 

Data on the removal of cows from the herd were downloaded from the German Database of Animal Movement (HI-Tier, Munich, Germany). Survival analysis was conducted via R package survival [19] both for life span as well as survival after the reporting of MAP-positive and MAP-negative test results. Survival of MAP-positive and MAP-negative cows, as well as of cows with or without MAP-specific antibodies, was calculated starting from the day of reporting test results of each year for each year. A Kaplan–Meier curve was generated for visualisation purposes. 

For the estimation of the 305-day fat-corrected milk yield, milk protein, and milk fat level, with the predictors FC status, parity, and year of testing (2012–2016), we used one multivariable linear regression model each. To meet all assumptions of the model, we restricted the data set to cows with a 305-day milk yield above 5250 kg, which resulted in 2312 observations. The four model assumptions, linearity, normality, homoscedasticity, and independence of residuals, were confirmed graphically. Additionally, the normality of the residuals was tested using the Shapiro–Wilk test.

## 3. Results

In 2022, after a 10-year period with consequent measures to control MAP, the dairy herd was certified as ‘non-suspect’ with respect to paratuberculosis, based on testing the individual faecal samples of all cows of the herd for MAP with negative results in the annual testing during three consecutive years. 

From 2012 to 2022, a total of 5299 individual faecal samples were tested. The prevalence of MAP shedders decreased from 17,83% to 0% in 2020, 2021, and 2022. In parallel, 4113 blood samples were analysed for MAP antibodies and the seroprevalence of MAP-antibody-positive cows decreased from 7.76% in 2012 to 1.45% in 2020. In total, 253 cattle were identified as MAP shedders. Mainly because of undesirable growth, 162 samples were not analysable for most of the years 2013 and 2017, where a high amount of mould spores was in the fodder and, subsequently, in the faeces, causing an unusually high amount of overgrowth. The number of high and very high shedders decreased over time, being ten in 2012, four in the years 2013 and 2014, and three animals in 2015. During nine years of ELISA testing, 156 cows were identified as MAP-antibody positive. Cohen’s kappa of test results from the faecal culture and MAP-antibody ELISA was ‘moderate’ in 2012 and decreased to ‘fair’ in the following years. In the years from 2016 to 2019, the kappa coefficient was calculated based on only one to seven MAP-positive animals, causing very large 95% confidence intervals (Table 1, Table 2 and Table 3).

**Figure 1:**
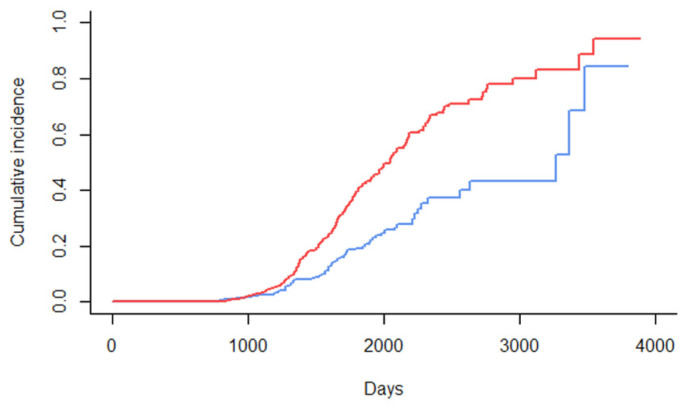
Cumulative incidence of new cases, as identified by faecal culture (red line) or MAP-antibody ELISA (blue line), in relation to the age of the cows (in days).

The cumulative incidence of new cases, as identified by FC or MAP-antibody ELISA in relation to the age of the cows, increased very slowly during the third year of living (730–1095 days) and afterwards in a nearly linear manner until the end of the seventh year (2555 days). At this age, nearly 70% of MAP-positive cows were identified by faecal culture and nearly 60% by ELISA (Figure 1).

Out of the 253 MAP shedders, 73 (29%) were five years or older when detected. During the first five years of control (2012-2016) 164 (71%) of the 231 MAP shedders were identified when they were 2-4 years old (Table 4).

**Figure 2:**
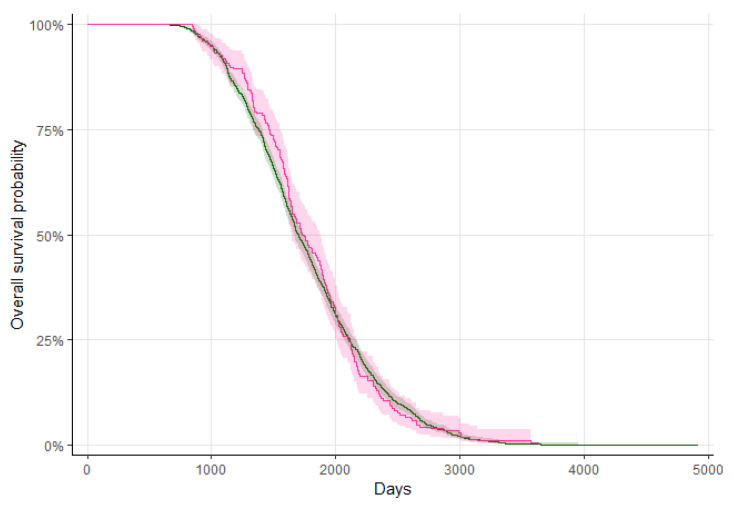
Overall average survival probability and confidence interval of MAP-negative (green line and shade) and MAP-positive (red line and shade) cows.

The overall average survival probability did not differ between MAP-negative and MAP-positive cows. Out of 1533 MAP-negative cows, 861 (56.2%) left the herd until 1800 days of living while the percentage of MAP-positive cows was very similar (52.9%, 110 of 208, Figure 2.

**Figure 3:**
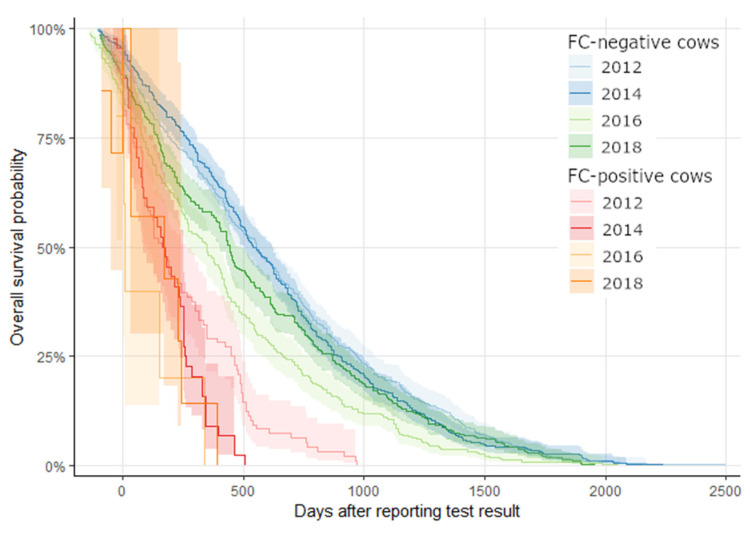
Survival rate and 95% confidence interval (shadow) of MAP-positive and MAP-negative cows after reporting the test result to the farmer after annual testing in 2012, 2014, 2016, and 2018.

Survival rate as calculated from the day of reporting the test result to the herd manager differed markedly for MAP positive and MAP negative cows, and for MAP positive cows between years of testing. In all year survival of MAP positives was shorter compared with the MAP negatives. In 2012, the first year of testing, with 96 MAP positive cows included in the survival analysis, 60 (62.5%) cows had left the herd until 300 days after reporting the test result. This percentage increased in the following years (e.g. in 2014: 35 out of 44 cows, 79.5%), and in 2016 and 2018 only one MAP positive cow was still in the herd at 300 days after reporting the test result. Only a minor percentage of MAP shedders was removed from the herd within 50 days after knowing that they are MAP positive, e.g. 27% in 2012 and 43.8% in 2018. This was due to the farm-specific strategy to keep shedders if their milk yield is acceptable, but not to rebreed them (Figure 3).

In 2012, four of five environmental samples taken from the walkways in the pens of the cows, the main alleyway to and the waiting pen in front of the milking parlour, the area in front of the calf igloos, and the sick cow pen were positive in FC. An additional sample taken by the sock sampling technique tested positive as well. The cultivation of liquid manure samples, which was carried out from 2012 to 2018 and in 2022, showed positive results until 2015. The direct PCR test, as applied during 2015–2018 and 2022, was only negative in 2022. The FC of biomass samples after fermentation always had negative results while the PCR was positive in 2015 and 2017 but not in 2022 (Table 5).

**Figure 4:**
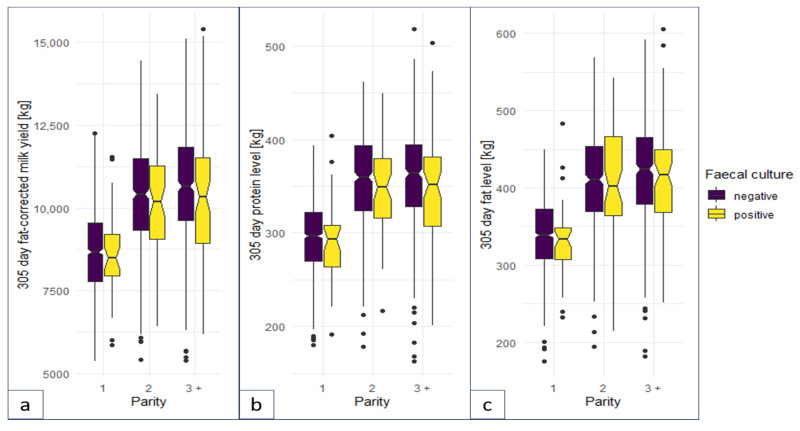
Boxplots for (**a**) 305-day fat-corrected milk yield, (**b**) 305-day milk protein, and (**c**) 305-day milk fat for faecal-culture-positive and -negative cows.

The multivariable linear regression model predicts a significantly decreased milk yield (305 days, fat corrected) and milk protein of FC-positive cows compared with FC-negative cows respecting the covariates parity and year of testing (Figure 4, Table 6, Table A1 and Table A2). The least-square means differed significantly by 267 kg for milk yield and 9 kg for milk protein, with no significant differences in milk fat (Table 7).

During 2012–2022, total costs of testing amounted to EUR 133,771.20. These costs were mainly generated by the individual FC (EUR 115,172.00; 86%, Table A3). As half of the FC and PCR testing costs were subsidised by the TPCP fund and the ELISA test was performed at the cost of the laboratory (additional test because of scientific interests), the costs remaining with the farmer were EUR 57,963.00. 

## 4. Discussion

This retrospective analysis and report described the progress of the successful control of paratuberculosis in a closed herd of dairy cows according to the TPCP. The whole process, from identifying the herd as affected with paratuberculosis with an estimated initial true prevalence of MAP shedders of 24.2% until certification as ‘non suspect of having paratuberculosis’, lasted 10 years. In the TPCP, cattle herds are certified as ‘non-suspect’ after three years without detection of MAP in the yearly testing of an individual sample of each cow for MAP by FC or PCR and without violating the rules on animal movements. Taking together the negative test results from repeated testing of the herd’s cows and the epidemiological aspects of the closing of the herd and the long-lasting certification period (three years), the probability of freedom from paratuberculosis for the study herd is assumed to be high; although, calculating the probability of freedom from paratuberculosis for a distinct cattle herd is subjected to further research. Considering the limited diagnostic sensitivity of faecal culture, we can state now that paratuberculosis was controlled to a level where MAP was not detectable anymore in repeated individual faecal samples from each cow. In light of the results achieved in other herds in the TPCP, the herd status ‘non-suspect of having paratuberculosis’ provides a high level of certainty that MAP is not present anymore at the farm. The results of testing liquid manure samples collected at the pipe from the barn to the biogas plant support this assumption. The farm is still monitored according to the TPCP and the results of the following years will clarify if the elimination of MAP from the farm was indeed successful, or not. 

The elimination of MAP from a cattle herd with a reasonable prevalence of MAP shedders was questioned during the last decades and elimination has not yet been demonstrated in any field studies [20,21]. Several longitudinal studies described the progress of paratuberculosis control in dairy herds to a certain level of prevalence reduction. For example, in seven dairy herds in Minnesota, the proportion of culturally MAP-positive animals fell from 10.5% to 5.6% and in nine herds in Wisconsin from 17.0% to 9.5% [22,23], providing evidence that it is possible to noticeably reduce the proportion of infected animals in dairy herds within a period of 5–7 years. Consequently, most of the control programmes worldwide have the objective of reducing prevalence and stamping out was the goal only in countries where the disease was very uncommon or absent, such as Sweden and Norway [3]. In contrast, in Thuringia, a federal state in the eastern part of Germany, the estimated true between-herd prevalence was approximately 50% in 2018 [24]. Nonetheless, a relevant percentage of the cattle farmers who enrolled in the TPCP claimed the elimination of MAP at the herd level is their goal. In 2020 a total of 58 farms with more than 18,000 cows were certified as ‘non-suspect’ regarding paratuberculosis [9], which corresponds to a rather high probability of freedom from paratuberculosis. Achieving this goal, even if the initial MAP shedder prevalence in the herd is high, is the peculiarity of this study. A similar success was reported for a goat herd [7] but, to the author’s best knowledge, not yet for a large commercial dairy herd. 

During the first 5 years of control (2012–2016), a sharp reduction in MAP shedder prevalence, as detected by FC, was achieved while, during the following year, a low-level shedder prevalence persisted. A similar sequence was observed in other herds, as reported elsewhere [8]. This long tailing-out is caused by the long-lasting pathogenesis of paratuberculosis where time from birth to detectable shedding can last up to 10 years and some cows are prone to become shedders and other cows control the infection [25]. In case of such a low shedder prevalence, it may be challenging to motivate farmers to maintain control measures until a high probability of freedom from paratuberculosis is achieved and not let things slide [8]. 

Compared with the detection of MAP shedders by FC, the MAP-antibody ELISA test identified far fewer infected animals during the first years of control and the true prevalence estimated from these data was remarkably lower, particularly during the first year of control. A relevant proportion of MAP shedders would not have been identified if the control strategy had relied on the ELISA test only. The long-time communicated failure of the test-and-cull approach in the control of paratuberculosis might be due to this low agreement between the FC and ELISA test while preferring the ELISA test because of economic reasons. Consequently, epidemiological models for control strategies that focused on the identification and removal of serological reactants did not, or only to a small extent, lead to a reduction in prevalence [6,26]. Therefore, the TPCP relies on the identification of MAP shedders by individual FC in affected herds that are in the control stage and do so in the future. A second reason for that decision was the limited specificity of the ELISA test. Although the German National Reference Laboratory published a substantial specificity for the ELISA test applied in this study (99.3%) [11], six cows (1.45%) tested positive in 2020 when no MAP shedder was detected by FC. This corresponds to the specificity of 97.7% of that test, as calculated in a field study in control program herds [27]. 

The kappa coefficients for the agreement of FC and ELISA were generally low, which is in line with the results of other studies [28,29]. The kappa decreased from 0.46 in 2012 to 0.33 in 2014 and 2015. In the first year of control with a relevant proportion of high shedders, the agreement was substantial because ELISAs have a higher sensitivity in high shedders compared with low shedders [27,30,31]. In the next years of our study (2013–2015), the agreement of FC and serological testing was diminished by the lowered detection rate of shedders by the ELISA tests, which is due to the association between the sensitivity of the ELISA and the stage of infection of the cattle tested [31]. Because the immune response to MAP is cell-mediated in the early stage of disease and humoral immunity follows in later stages, the negative ELISA test outcome is likely a result of the missing humoral immune reaction and not a weakness of the ELISA test itself [32]. 

We observed that the probability of identifying a MAP shedder by the first positive test result of either the FC or ELISA increases in an almost linear manner with age. This is in line with the results of a Danish study to detect ‘infection’ [33]. In their study, the authors calculated a greater sensitivity for the ELISA than for the FC, to some degree at the expense of specificity. The test used in our ELISA study had a good specificity of 99.3% and a sensitivity of 52.8% [11], which is lower than the sensitivity of the FC performed with Herrold’s Egg Yolk Medium [34] and in line with a Canadian study [35]. In our study, the higher sensitivity of FC contributes to an earlier detection of a high proportion of test positives in younger age. Against the background, that a considerably higher number of animals were MAP-positive in FC than MAP-antibody-positive in ELISA (253 versus 156) in parallel testing, the use of FC had a remarkably higher potential to identify MAP-infected animals. Considering this advance in test performance, the apparent disadvantages of FC were compensated. First of all, the cost effectiveness of FC was doubted [36]. Another disadvantage is the long lag of time until a FC test result can be reported (up to 12 weeks). This delay was suspected to trigger the spread of the infection [37]. Our results suggest that it is more important to detect a high percentage of MAP-shedding animals and to manage them accordingly rather than leave them undetected in the herd. Only 73 (29%) of the MAP shedders were five years or older when detected (Table 4). This includes also 20 MAP shedders that were at this age at first testing in 2012. This is consistent with a similar age-related analysis in Danish cows where nearly 27% of FC positives were 5 years or older when they were positively tested for the first time [33]. Nonetheless, during the first five years of control (2012–2016), most of the MAP shedders were younger than 5 years old when identified. During the last two years of control (2018–2019), none of the younger cows (<4 years) were identified as MAP shedders; however, eight cows at higher ages tested positive for the first time and they had tested MAP-negative for several years in advance. Therefore, multiple testing of each animal remains necessary to identify all MAP shedders when elimination of the infectious agent is the goal. Obviously, the most pronounced drop in shedder prevalence was observed after the fourth and the eighth year of control, suggesting an association with birth cohorts of calves born after establishing hygienic measures or the generation interval of dairy cows of approximately three lactations. This observation is specific to the study herd and the interpretation is hypothetical; it should not be generalized and implies further research. 

As shown by the overall average survival probability that was not different between MAP-negative and MAP-positive cows, the culling strategy of the herdsman was moderate. Only the high and very high shedders identified in the first two years of the control program were aggressively culled, i.e., without delay, after getting the test result. As demonstrated by modelling studies, the contact of susceptible adult animals in the milking herd compartment with high-shedding animals is one of the most important factors for the increase in prevalence among adult cows [21,38]. During the first years of control, calving was allowed for all other MAP-positive cows (moderate and low shedders). They were not rebred and milked for the next lactation and then removed and all the MAP-positive cows received a flag on the culling list and were prioritized for voluntary culling. This approach is consistent with the outcome of another modelling study that recommends an annual test frequency, culling of moderate and high shedders, and early culling of high shedders [39]. In the following years, when the number of new MAP-positive cows decreased substantially, low shedders were also removed as soon as possible (Figure 3, red lines). This approach was consistent with the outcome of a modelling study, which showed that the culling of infectious animals with a testing interval longer than 6–12 months is generally not effective in controlling MAP [37]. Since 2016 only single-digit numbers of new MAP positives were identified, which is less than 2% of the herd’s cows and only a small fraction of the target culling rate of 25%. The high impact of culling positive cows was demonstrated by a Danish modelling study where within-herd prevalence could be reduced to zero only if that measure was included [36]. Similarly, in another modelling study, the culling of all MAP positives, as detected by FC or PCR, was the most effective approach to reduce prevalence within 5 years [40]. To the best knowledge of the authors, our study is the first field report that substantiates the outcome of these models with real-world data, which is an important step to further improve models [41].

For a long time, improving hygiene in a farm was considered the most relevant approach to minimize the spread of MAP within a herd and test-and-cull strategies without closing infection routes, mainly those from adult cows to their calves, were found to be ineffective in reducing prevalence by simulation models [42]. A review of present knowledge regarding the relevance of distinct hygienic measures revealed that minimizing the contact of calves with the faeces of adults is most important [43]. Our study showed, that a meaningful selection of measures that can be kept each day was a practical and effective approach. Measures that are simple to implement into daily practice are effective, such as a separate calving pen for MAP-shedders and the separation of calves from their dam immediately after calving. At least during the daytime, calves were separated within 1 to 1.5 h after calving from their dam. During that time, cows were allowed to lick their calves but calves were not allowed to suckle. For calvings, during night time (10 p.m. to 5 a.m.), it was not possible to guarantee this procedure. In light of this gap and to mitigate the relevant risk of intrauterine transmission [44], in 2018, the farmer decided to breed all heifers that originate from MAP-positive cows to terminal sire and to sell the calves for fattening. The non-appearance of new MAP shedders in the following years suggests that this measure was effective in mitigating the remaining risk of persistent MAP infection resulting from the above-mentioned gaps in hygiene management. 

Although a systematic review did not identify studies that proved a significant risk-mitigating effect of colostrum management [43], colostrum management is among the most popular measures to limit the transmission of MAP to young calves [36]. In the study herd, colostrum was carefully milked using a clean separate milking machine to avoid faecal contamination strictly. A separate room for preparing milk for calves and cleaning the teat buckets was established and used only by calf carers. Entry with dirty boots was strictly prohibited. In addition, in-line feeding of first-feeding colostrum (i.e., only from the own dam) was applied whenever possible. Colostrum for later feedings originated only from MAP-negative cows and non-saleable milk from MAP-positive cows (i.e., milk of 1–5 days in milk) was disposed of. We are not able to distinguish which of these measures regarding colostrum supply was effective for the control of MAP in our study herd. The evidence for the effectiveness of colostrum management in the control of MAP from real-world risk factor studies is weak and based on a small number of studies. Feeding colostrum from one cow to multiple calves was slightly associated (OR = 1.10) with classifying cows as infected with MAP based on testing positive on FC or serum ELISA [45] and, as revealed by a survey study in Spain, utilisation of colostrum from cows with a previous positive MAP diagnosis was identified as a risk factor for a herd to be tested as seropositive [46]. In contrast, other studies did not identify an association between colostrum management and the ELISA status of cows or herds, respectively [47,48]. 

Environmental faecal samples, including samples of liquid manure, are widely established as an easy-to-use diagnostic tool to identify MAP-positive herds [12,49,50] and turned out to be a convenient tool to monitor the progress of MAP shedder prevalence reduction within a dairy herd in our study. Liquid manure samples, as collected in annual intervals, showed MAP-positive results during the first years of the control program when the apparent MAP shedder prevalence was above 5%. This is consistent with the results of a former study of 77 dairy herds where testing a liquid manure sample with FC was proven to detect a herd with an apparent within-herd prevalence of approximately 6.5% with a probability of 90% as MAP positive [12]. During the following years, when this prevalence dropped below 2%, the FC of liquid manure samples was consistently negative and only MAP DNA was detectable (Table 4). As previously shown in a larger study [51], mesophilic fermentation in the biogas plant was able to reduce viable MAP as detectable by FC to a non-detectable level, even in the first years of this study when the shedder prevalence was high. This was important information for the farmer as the biomass substrate after fermentation and storage was used as fertilizer and a re-introduction of MAP into the herd together with fodder plants should have been prevented. The detection of MAP DNA by PCR showed a positive signal, even in the years 2015 and 2017 with low MAP prevalence but not in 2022, when no MAP shedder had been detected for 3 consecutive years. Therefore, this approach seems to be a sensitive tool to prove the success of the herd-level control of MAP and a high probability of freedom from paratuberculosis. Unfortunately, the testing of liquid manure and biomass was not performed each year because the PCR test was not established before 2015 and, during 2019–2021, sampling was not performed. More studies are necessary to substantiate these results. 

In concordance with the results of other studies [2], the 305-day fat-corrected milk yield of MAP shedders was lower than those of the MAP-negative herd mates during the first five years of control (2012–2016). The difference of least-square means of 267 kg was less than the reduction predicted by a previously published meta-analysis [52], indicating a well-managed feeding of cows in the study herd. The variation in milk yield throughout the study years and between primiparous and multiparous cows was considered by including both variables as covariables in the model. Differences in milk protein and milk fat between MAP-positive and MAP-negative cows were neglectable; however, for milk protein, a significant difference was detected. This is consistent with the results of previous studies where milk protein was lowered in MAP-positive cows [2]. Therefore, the economic effect on milk production resulting from paratuberculosis control was very limited in our study herd. Nonetheless, the farmer observed better fitness in multiparous cows and a more pronounced drop in milk of MAP shedders of second and higher parity. This was worth spending EUR 57,963 on paratuberculosis control during 11 years, which is approximately EUR 11.70 per cow and year. In general, the farmer and herdsman of the study herd were very committed to paratuberculosis control and the program was successful on their farm. It took approximately one decade to achieve a high probability of freedom from paratuberculosis. Their activities were supported by financial aid from the regional Animal Disease Fund that covered half of the diagnostic costs and provided counselling by veterinary specialists at regular intervals. Furthermore, since 2016 (achievement of Level 4 of the TPCP), a subsidy of EUR 200 per cow was paid for each MAP shedder that was removed within one month after getting the test result (expect pregnant cows: one month after calving). Multiple testing by faecal culture in annual intervals combined with the removal of MAP shedders from the herd at a good pace and hygienic measures to reduce the infection of young calves were proven effective in reducing within-herd transmission. The removal of MAP shedders was accelerated during the control program in the study farm. This measure contributed to a reduction in the transmission risk by adult-to-adult contacts within the milking herd. This transmission route is controversially discussed. Modelling studies suggested that contact of adult cows with high-shedding herd-mates increased adult shedder prevalence [21,38]. In contrast, another modelling study showed that the effect of super-shedders on MAP transmission is limited. In their study, a thousand-fold increase in individual bacterial load was associated with only a two-fold to three-fold increase in infectiousness [53]. Nonetheless, the potential transmission risk by adult-to-adult contacts underlines the importance of removing all MAP shedders, and not only high shedders, in a timely manner. The low level of agreement between MAP detection in faecal samples compared with MAP antibody detection in blood samples did not allow for a substitution of FC by ELISA to identify high-risk animals. In a relevant manner, the better specificity and slightly higher sensitivity of FC compared to the ELISA test contribute to an earlier and more accurate detection of MAP shedders, which are most important for within-herd transmission. The farmer was willing to cope with the slightly higher effort for faecal sampling compared to blood sampling and the diagnostic costs were justified by the success of the control and the elimination within a reasonable period. A prerequisite for the success of the control in a region with a high between-herd prevalence of approximately 50% [24] was closing the herd and restocking by youngstock grown on the own farm. Financial aid and specialist advice were very acknowledged by the farmer and contributed to the success of the control. Nonetheless, the commitment of the farmer was decisive for the success.

The originality of our study is the follow-up of paratuberculosis control in a dairy herd to a level where MAP is not detectable anymore over a period of three years, representing a high probability of freedom from paratuberculosis and suggesting the successful elimination of infectious agents from that herd. During the certification period of three years, individual faecal samples of each cow were sampled in annual intervals and tested with only negative results for MAP by FC. Additionally, in our study herd at the end of the certification period, neither viable MAP nor MAP DNA were detectable in samples of liquid manure and of biomass from the run-off of the biogas, indicating that the infectious agent was not detectable anymore in that herd. In parallel, each cow of the herd was individually tested for MAP-specific antibodies in annual intervals as long as they were kept in the herd. Additionally, milk recording data were available for a high proportion of the cows and animal movement data were gathered. This intensive diagnostic approach, together with a follow-up period of 10 years, is the strength of our study and its unique feature. Nonetheless, some weaknesses of this study should be mentioned. During the last years of control (2016–2019), only a small number of MAP-positive animals was available, causing high uncertainty in the calculation of the kappa value reflected by the large confidence interval for these years. However, the results of several larger studies support the main outcome that the level of agreement between the MAP detection by FC and the MAP-antibody ELISA is low, particularly for low shedders [25,32,54]. Although there were no complete data regarding MAP detection in liquid manure and biomass from the biogas plant, these results were consistent with the results of a larger study [51] and were an extension of the diagnostic processing according to the TPCP, which was not necessarily requested in each year. Most importantly, this report describes the process of paratuberculosis control in one specific study herd and is not generalisable. Each dairy has its own particularities and the perceptions toward the implementation of on-farm paratuberculosis control strategies may differ [55]. In our study, the farmer and the herdsman were highly motivated to struggle through the tribulations of paratuberculosis control, which is specific but not uncommon among Thuringian dairy farmers. A former study investigating the attitudes of farmers toward paratuberculosis control in that region identified a high proportion of cattle farmers supporting the activities of paratuberculosis control [56]. For those who are interested in paratuberculosis control but still undecisive, this report may be a good example showing that the disease can be controlled and even that the elimination of the infectious agent might be an achievable goal. 

## 5. Conclusions

Within an acceptable period of approximately ten years, control of paratuberculosis in a closed dairy herd can achieve a probability of freedom from paratuberculosis that might suggest a successful elimination of the infectious agent. MAP detection in faecal samples is an appropriate diagnostic approach to detect MAP shedders, which can only be partly replaced by testing for MAP-specific antibodies. MAP shedders must be removed from the herd in a timely manner to reduce the risk of within-herd transmission between cows and from cows to calves. Keeping calves outside the premises of adult cows and establishing hygienic measures to minimize the MAP exposure of calves is crucial and must be maintained at all times. The general MAP load within the farm can be monitored by testing liquid manure samples for viable MAP or MAP genome segments. The Thuringian Paratuberculosis Program provides an effective approach to control paratuberculosis and adequate logistic and financial support for farmers to achieve a high probability of freedom from paratuberculosis for cattle herds.

## Figures and Tables

**Table 1:** Results of annual testing of individual faecal samples for *Mycobacterium avium* subsp. *paratuberculosis* by bacteriological cultivation during 2012–2022 and resulting MAP shedder prevalence for each year.

	Faecal Culture								
Year	Totally Tested [*n*]	Positively Tested [*n*]	Negatively Tested [*n*]	Not Valid [*n*]	Apparent Prevalence [%]	True Prevalence [%]	Confidence Interval [%]		
2012	601	107	493	1	17.83	24.20	20.20	–	28.50
2013	614	50	501	63	9.07	12.30	9.30	–	15.90
2014	522	45	477	0	8.62	11.70	8.70	–	15.30
2015	579	36	543	0	6.22	8.40	6.00	–	11.30
2016	348	5	338	5	1.46	2.10	0.70	–	4.50
2017	435	2	340	93	0.58	0.90	0.10	–	2.70
2018	431	7	424	0	1.62	2.30	0.90	–	4.40
2019	413	1	412	0	0.24	0.40	0.00	–	1.70
2020	478	0	478	0	0.00	Not calculated			
2021	432	0	432	0	0.00				
2022	446	0	446	0	0.00				
Total	5299	253	4884	162					

**Table 2:** Results of annual testing of blood samples for *Mycobacterium avium* subsp. *paratuberculosis* antibodies by ELISA during 2012–2020 and resulting prevalence of MAP-antibody-positive cows for each year.

	MAP-Antibody ELISA								
Year	Totally Tested [*n*]	Positively Tested [*n*]	Negatively Tested [*n*]	Suspicious [*n*]	Apparent Prevalence [%]	True Prevalence [%]	Confidence Interval [%]		
2012	490	38	451	1	7.76	12.80	9.00	–	17.50
2013	526	31	488	7	5.89	9.50	6.20	–	13.50
2014	488	38	446	4	7.79	13.50	9.00	–	17.70
2015	481	11	470	0	2.29	3.10	1.10	–	6.10
2016	437	10	424	3	2.29	3.10	1.00	–	6.30
2017	435	10	425	0	2.30	3.10	1.00	–	6.20
2018	433	8	424	1	1.85	2.30	0.50	–	5.20
2019	410	4	405	1	0.98	1.00	0.10	–	3.20
2020	413	6	407	0	1.45	1.70	0.20	–	4.30
Total	4113	156	3940	17	Not calculated				

**Table 3:** Cohen’s kappa coefficient (κ), as calculated for the detection of paratuberculosis by the testing of blood samples for MAP antibodies and testing of individual faecal samples for MAP by faecal culture for each year of testing.

Year	Cohen’s Kappa Coefficient			
	κ	95% CI (κ)		
2012	0.46	0.36	–	0.58
2013	0.29	0.15	–	0.44
2014	0.33	0.19	–	0.48
2015	0.33	0.16	–	0.51
2016	0.42	0.09	–	0.74
2017	0.19	−0.14	–	0.52
2018	0.12	−0.12	–	0.36
2019	0.40	−0.14	–	0.94

**Table 4:** Number of faecal-culture-positive cows by age, as detected in each year of testing.

Age [Years]	2012	2013	2014	2015	2016	2017	2018	2019
2	21	12	3	3	2	0	0	0
3	23	8	16	14	0	1	0	0
4	34	12	6	10	0	0	3	0
5	10	13	13	5	1	1	3	0
6	5	2	4	2	2	0	0	0
7	1	1	2	1	0	0	0	1
8	0	1	0	0	0	0	1	0
9+	4	0	0	0	0	0	0	0

**Table 5:** Detection of viable MAP by bacteriological cultivation and MAP DNA fragments by polymerase chain reaction in liquid manure samples before fermentation in a biogas plant and in biomass samples after fermentation in a biogas plant.

Date of Sampling	Liquid Manure Samples before Fermentation				Biomass Samples after Fermentation			
	Culture	Growth	PCR	Ct-Value	Culture	Growth	PCR	Ct-Value
6 March 2012	Pos.	moderate	Not applied		Neg.	-	Not applied	
21 September 2012	Pos.	low	Not applied		Neg.	-	Not applied	
15 May 2013	Pos.	low	Not applied		Neg.	-	Not applied	
4 March 2014	Pos.	moderate	Not applied		Neg.	-	Not applied	
27 April 2015	Pos.	low	Pos.	36.6	Neg.	-	Pos.	36.6
1 March 2016	Neg.	-	Susp.	39.3	Neg.	-	Neg.	-
1 March 2017	Neg.	-	Pos.	36.7	Neg.	-	Pos.	37.0
1 March 2018	Neg.	-	Not applied		Neg.	-	Not applied	
22 February 2022	Neg.	-	Neg.	-	Neg.	-	Neg.	-

**Table 6:** The prediction of milk yield (305 days, fat corrected) for faecal-culture-positive and -negative cows and covariables of parity and the year of testing by means of a multivariable linear regression model.

Predictors		Estimates	Confidence Interval	*p*-Value	Wald-Test *p*-Value
(Intercept)		8507.04	8356.72–8657.35	<0.001	
Faecal culture	negative	reference			
	positive	−266.57	−511.30–−21.84	0.033	
Parity	1	reference			
	2	1721.70	1571.64–1871.77	<0.001	<0.001
	3+	1918.84	1763.10–2074.58	<0.001	
Year	2012	reference			
	2013	188.55	−2.02–379.11	0.052	0.001
	2014	303.87	111.51–496.23	0.002	
	2015	66.17	−122.95–255.30	0.493	
	2016	266.11	46.03–486.20	0.018	
Observations		*n* = 2312			
R2/R2 adjusted		0.275/0.272			

**Table 7:** Least-square means (±standard error) of milk yield (305 days, fat corrected), milk protein, and milk fat for MAP-negative and MAP-positive cows.

	Milk Yield [kg] (SE)		Milk Protein [kg] (SE)		Milk Fat [kg] (SE)	
Parity	FC (+)	FC (−)	FC (+)	FC (−)	FC (+)	FC (−)
1	8405 (130.4)	8672 (45.7)	286 (4.01)	295 (1.54)	333 (4.86)	339 (1.7)
2	10,127 (128.4)	10,394 (62.1)	348 (3.94)	358 (1.91)	404 (4.78)	409 (2.31)
3+	10,324 (126.0)	10,591 (65.5)	348 (3.87)	358 (2.01)	415 (4.69)	420 (2.44)

## Data Availability

Data are contained within the article (Table 1, Table 2, Table 3, Table 4, Table 5, Table 6 and Table 7, Figure 1, Figure 2, Figure 3 and Figure 4, Table A1, Table A2 and Table A3). Details of laboratory analysis are available upon request.

## Appendix A

**Table A1:** The prediction of milk protein (305 days) for faecal-culture-positive and -negative cows and covariates of parity and the year of testing by means of a multivariable linear regression model.

Predictors		Estimates	Confidence Interval	*p*-Value	Wald-Test *p*-Value
(Intercept)		293.43	288.82–298.05	<0.001	
Faecal culture	negative	reference			
	positive	−9.16	−16.68–−1.64	0.017	
Parity	1	reference			
	2	62.63	58.02–67.24	<0.001	<0.001
	3+	62.56	57.78–67.35	<0.001	
Year	2012	reference			
	2013	7.97	2.11–13.82	0.008	<0.001
	2014	4.15	−1.76–10.06	0.169	
	2015	−7.14	−12.95–−1.32	0.016	
	2016	2.56	−4.21–9.32	0.458	
Observations		*n* = 2312			
R^2^/R^2^ adjusted		0.321/0.319			

**Table A2:** The prediction of milk fat (305 days) for faecal-culture-positive and -negative cows and covariates of parity and the year of testing by means of a multivariable linear regression model.

Predictors		Estimates	Confidence Interval	*p*-Value	Wald-Test *p*-Value
(Intercept)		337.71	332.11–343.31	<0.001	
Faecal culture	negative	reference			
	positive	−5.63	−14.75–3.49	0.226	
Parity	1	reference			
	2	70.88	65.29–76.47	<0.001	<0.001
	3+	81.74	75.94–87.54	<0.001	
Year	2012	reference			
	2013	7.67	0.57–14.77	0.034	0.001
	2014	1.97	−5.20–9.14	0.590	
	2015	−6.82	−13.87–0.22	0.058	
	2016	1.22	−6.98–9.42	0.770	
Observations		*n* = 2312			
R^2^/R^2^ adjusted		0.322/0.320			

**Table A3:** Total costs of testing in EUR, as calculated from the number of samples tested during each year and the cost per test in the respective year.

ELISA Test	Individual Faecal Culture	Culture of Environ-mental Samples ^1^	PCR of Environmental Samples ^1^	Total	
2012	1954.65	10,620.00	198.00	0.00	12,772.65
2013	1900.70	11,016.00	36.00	0.00	12,952.70
2014	1904.85	9360.00	36.00	0.00	11,300.85
2015	1809.40	10,368.00	54.00	64.00	12,295.40
2016	1813.55	6920.00	40.00	60.00	8833.55
2017	1805.25	8660.00	40.00	60.00	10,565.25
2018	2294.90	10,725.00	50.00	0.00	13,069.90
2019	2173.00	10,325.00	0.00	0.00	12,498.00
2020	2188.90	11,774.00	0.00	0.00	13,962.90
2021	0.00	12,528.00	0.00	0.00	12,528.00
2022	0.00	12,876.00	58.00	58.00	12,992.00
Total	17,845.20	115,172.00	512.00	242.00	133,771.20

^1^ includes the costs of testing environmental samples, liquid manure samples, and biomass samples after fermentation in the biogas plant.
